# From the President’s Desk – Part 3, 2022

**DOI:** 10.4102/safp.v64i1.5546

**Published:** 2022-05-19

**Authors:** Robert Mash

**Affiliations:** 1 Division of Family Medicine and Primary Care, Faculty of Medicine and Health Sciences, Stellenbosch University, Cape Town, South Africa; 2 South African Academy of Family Physicians, Cape Town, South Africa

This regular feature is intended to keep you updated with the work of the South African Academy of Family Physicians (SAAFPs). At the time of writing this editorial, the new national position paper on the contribution of family physicians to the South African health system was just published.^1^ I encourage you to read it.

We want this new position paper to influence human resources for health policy at provincial and national levels and recommend you to share or use it in your conversations with policymakers. Our medium-term goal is for a family physician in each district hospital and community health centre or sub-district, throughout the country, in addition to those appointed to district clinical specialist teams.^1^ The numbers needed to achieve this and to increase the required number of posts for both registrars and family physicians have been analysed. There will be a meeting with the national council of the SAAFP to strategise how we can best disseminate the position paper and its key messages to our stakeholders.

The position paper also speaks to the issues in the private sector and the need to properly recognise the family physician’s scope of practice and to provide appropriate remuneration.^1^ The need for recognising the unique features of private general practice is also mentioned, where you may have specialist family physicians and non-specialist general practitioners sharing one practice. We have been engaging with Healthman to take up these issues with the Board of Healthcare Funders and other stakeholders. In order to fully engage them, a private family physician forum within the SAAFP needs to be created and Dr Sheena Mathew is leading this process. It will also be necessary for the members of this group to contribute financially to the additional cost of contracting with Healthman. If you are in the private sector and wish to join this forum, please contact the SAAFP office (admin@saafp.org)

I would like to remind you that the National Family Practitioners Conference is planned for 19–20 August 2022 at the Lagoon Beach Hotel in Cape Town. The website is open for both abstracts and registrations. The theme of the conference is ‘Bouncing back – resilience in the face of change’. Please visit the website https://www.saafpcongress.org.za/ to view the exciting programme, with 8 plenary talks, 16 workshops and seminars, 100 oral or poster presentations, and additional pre-conference and industry-related events. We plan a 1-day pre-conference workshop on point-of-care ultrasound. If you have recently completed a research study, then please submit your abstract and we hope that everyone will register and enjoy the conference. We plan that most people will come face-to-face, but there will also be a virtual attendance option.

Another exciting development is the launch of a nationwide e-portfolio for the training of registrars in family medicine. The SAAFP coordinates access to the e-portfolio for all training programmes. The e-portfolio has been designed in SCORION software with Parantion, a company that has an extensive experience of supporting workplace-based learning and training. This e-portfolio will improve the quality of clinical training in the workplace, enable more quality assurance of training and supervision, and streamline the entry into the national fellowship examination. It will also be possible in the future to easily include assessment of the portfolio in the examination itself.

Other initiatives include the launch of a new continuing professional development (CPD) short course on telehealth that has been designed by Prof. Selma Smith. This adds to our existing short courses on brief behaviour change counselling and pregnancy-induced hypertension.

Our *South African Family Practice* journal has also appointed three new editors to improve the capacity of the journal and enhance the use of social media to disseminate the content. We welcome Ramprakash Kaswa from Walter Sisulu University, Shane Murphy from University of the Witwatersrand and Arun Nair from University of the Free State. Finally, our administrator Ms Janine Koeries is moving on, and the author would like to thank her for the valuable contribution. She will be replaced by Ms Lucille Boshoff.

Having shared with you the many ways in which the SAAFP is representing your interests as a family physician and meeting the needs of family doctors, I encourage you to become a member if you have not already done so. Please visit our website to find out more: https://saafp.org/.
